# Microbiota of Peri-Implant Healthy Tissues, Peri-Implant Mucositis, and Peri-Implantitis: A Comprehensive Review

**DOI:** 10.3390/microorganisms12061137

**Published:** 2024-06-02

**Authors:** Federica Di Spirito, Francesco Giordano, Maria Pia Di Palo, Francesco D’Ambrosio, Bruno Scognamiglio, Giuseppe Sangiovanni, Mario Caggiano, Roberta Gasparro

**Affiliations:** 1 Department of Medicine, Surgery and Dentistry, University of Salerno, Via S. Allende, 84081 Baronissi, Italy; frgiordano@unisa.it (F.G.); mariapia140497@gmail.com (M.P.D.P.); s.bruno2949@gmail.com (B.S.); gsangiovanni7@gmail.com (G.S.); macaggiano@unisa.it (M.C.); 2 Department of Neuroscience, Reproductive Science and Dentistry, University of Naples Federico II, 80131 Naples, Italy; roberta.gasparro@unina.it

**Keywords:** oral health, peri-implant health, peri-implant disease, peri-implant mucositis, peri-implantitis, dental implants, microbiota, dysbiosis, periodontitis

## Abstract

Understanding the microbiological profiles of peri-implant conditions is crucial for developing effective preventive and therapeutic strategies. This narrative review analyzes the microbial profiles associated with healthy peri-implant sites, peri-implant mucositis, and peri-implantitis, along with related microbiological sampling and analyses. Healthy peri-implant sites are predominantly colonized by *Streptococcus*, *Rothia*, *Neisseria*, and *Corynebacterium* species, in addition to Gram-positive cocci and facultatively anaerobic rods, forming a stable community that prevents pathogenic colonization and maintains microbial balance. In contrast, peri-implant mucositis shows increased microbial diversity, including both health-associated and pathogenic bacteria such as red and orange complex bacteria, contributing to early tissue inflammation. Peri-implantitis is characterized by even greater microbial diversity and a complex pathogenic biofilm. Predominant pathogens include *Porphyromonas gingivalis*, *Tannerella forsythia*, *Treponema denticola*, *Fusobacterium nucleatum*, and unique species like *Filifactor alocis* and *Fretibacterium fastidiosum*. Additionally, less common species such as *Staphylococcus* and *Enterobacteriaceae*, contributing to disease progression through biofilm formation and increased inflammatory response, along with *EBV* and human cytomegalovirus with a still not defined role, and *Candida albicans* contribute to disease progression through biofilm formation, immune modulation, and synergistic inter-kingdom interactions. Future research should standardize diagnostic criteria, employ advanced molecular techniques, integrate microbial data with clinical factors, and highlight inter-kingdom interactions.

## 1. Introduction

Dental implants have become an indispensable tool in modern dentistry, providing a highly effective solution for tooth replacement [1]. However, despite the notable success rates—estimated at 90% over 5 and 10 years—peri-implant diseases remain a significant challenge [2].

Peri-implant health is characterized by the absence of clinical signs of inflammation, such as erythema, bleeding on probing, swelling, and suppuration. Peri-implant tissues in health typically include a well-adapted peri-implant mucosa, which comprises a connective tissue core covered by either keratinized or non-keratinized epithelium. Histologically, the peri-implant mucosa averages 3 to 4 mm in height and includes a junctional epithelium facing the implant surface. The connective tissue adjacent to the implant is relatively free of inflammatory cells and is in direct contact with the implant surface. The bone surrounding the implant shows a high degree of osseointegration, with most of the intrabony portion of the implant in contact with mineralized bone [3,4,5].

Peri-implant mucositis is characterized primarily by the inflammation of the peri-implant mucosa without the accompanying bone loss seen in peri-implantitis. Clinically, it is identified as bleeding on gentle probing, often accompanied by erythema, swelling, and sometimes suppuration. Increased probing depth may occur due to swelling or decreased tissue resistance. Strong evidence supports plaque as the main etiological factor for peri-implant mucositis, similar to gingivitis in natural teeth. The condition is reversible with appropriate plaque control measures, and there is limited evidence for non-plaque-induced peri-implant mucositis. Histologically, peri-implant mucositis shows a well-defined inflammatory infiltrate composed of vascular structures, plasma cells, and lymphocytes lateral to the junctional epithelium, without extending into the supracrestal connective tissue zone [3,4,6]. Peri-implant mucositis is considered a reversible condition if detected early and managed appropriately. However, if peri-implant mucositis is left untreated, it can progress to peri-implantitis, where the inflammatory response extends into the supporting bone, leading to bone resorption.

Peri-implantitis is a more advanced pathological condition involving both the inflammation of the peri-implant mucosa and progressive loss of supporting bone [4,7]. Clinically, it is characterized by signs of inflammation such as bleeding on probing, suppuration, increased probing depths, and the recession of the mucosal margin, along with radiographic evidence of bone loss compared to previous assessments. Peri-implantitis lesions extend apically from the junctional epithelium and are characterized by dense inflammatory infiltrates, including plasma cells, macrophages, and neutrophils. Indeed, the pathogenesis of peri-implant mucositis and peri-implantitis involves an inflammatory response to bacterial biofilm accumulation. The initial host response includes the recruitment of neutrophils and the production of pro-inflammatory cytokines, such as interleukin-1β (IL-1β) and tumor necrosis factor-alpha (TNF-α). This localized inflammatory response aims to control the bacterial infection but can result in tissue damage if the biofilm is not adequately controlled [3,6]. The chronic inflammation associated with peri-implantitis is characterized by a shift from a neutrophil-dominated response to a more chronic inflammatory infiltrate that includes macrophages, lymphocytes, and plasma cells. These cells produce a range of pro-inflammatory mediators and enzymes, such as matrix metalloproteinases (MMPs), which degrade the extracellular matrix and bone [3,6].

Along with additional risk factors, including smoking, a history of periodontitis, genetic predisposition, systemic conditions such as diabetes [8], and inadequate oral hygiene practices, and contributing factors, such as the presence of residual cement from prosthetic reconstructions [9] and biomechanical overload on the implant [3,5], the primary etiological factor for both peri-implant mucositis and peri-implantitis is microbial biofilm accumulation on implant surfaces [3,6].

Recent advances in microbiological techniques, particularly next-generation sequencing (NGS), have provided new insights into the microbial communities associated with peri-implant diseases. These technologies allow for a comprehensive analysis of the microbiota, identifying both cultivable and non-cultivable species and revealing the complexity and diversity of the microbial communities involved.

Historically, the microbiological analysis of peri-implant diseases relied on culture-based methods, which are limited by the inability to cultivate many oral microorganisms. Newer methods, such as 16S ribosomal RNA (rRNA) sequencing and shotgun metagenomics, have overcome these limitations, providing a more detailed and accurate picture of the peri-implant microbiota. These methods have shown that peri-implantitis is associated with a diverse and complex microbial community, distinct from that of healthy peri-implant sites and periodontal diseases.

Understanding the microbiological profiles of these conditions is crucial for developing effective preventive and therapeutic strategies. Therefore, the aim of this narrative review was to analyze the microbial profiles associated with healthy peri-implant, peri-implant mucositis, and peri-implantitis sites and the related microbiological sampling and analyses.

## 2. Microbiological Sampling and Analysis of Peri-Implant Microbiota

Microbiological sampling is a fundamental process in understanding the composition and behavior of microbial communities, particularly in clinical contexts such as peri-implantitis. Effective sampling techniques are essential to accurately capture the microbial populations involved, preserving their integrity for subsequent analysis [10]. Following sample collection, the next step is the analysis of these samples to identify and quantify the microbial communities present. Various techniques are employed in microbiological analysis, each with distinct strengths and limitations, as discussed below [10].

Interpreting microbiological data requires robust statistical analysis to discern significant patterns and correlations. Statistical methods such as multivariate analysis, principal component analysis, and clustering techniques are commonly used to analyze complex microbiological datasets. 

These methods help identify relationships between microbial communities and clinical parameters, providing insights into the mechanisms underlying peri-implantitis and its treatment [11]. Statistical analysis is crucial for making sense of the large datasets generated by advanced molecular techniques, ensuring that findings are scientifically valid and clinically relevant. Indeed, a critical aspect of microbiological analysis is correlating findings with clinical outcomes. This involves comparing microbial data with clinical parameters such as probing depth, attachment loss, and radiographic bone levels. Studies have shown that successful peri-implantitis treatment is often associated with a reduction in pathogenic bacteria and an increase in beneficial microbial species [12]. Understanding these correlations can guide the development of targeted therapies aimed at restoring a healthy microbial balance.

Despite advances in microbiological analysis, several limitations persist. These include the inherent variability in sampling techniques, the influence of host factors on microbial communities, and the potential for laboratory contamination. Additionally, the complexity of the oral microbiome and its interactions with the host immune system pose challenges for interpretation. Future research should focus on standardizing sampling and analysis protocols, exploring the role of the host immune response, and developing novel therapeutic strategies based on microbiome modulation [13].

### 2.1. Microbiological Sampling at Peri-Implant Sites

The efficacy of microbiological studies relies significantly on the robustness of sample collection techniques. In the context of peri-implantitis, several methods are commonly employed, including swabbing, scraping, and the use of paper points. Swabbing involves using sterile swabs to collect biofilm from the peri-implant pockets. This method is advantageous due to its non-invasive nature and effectiveness in collecting surface microbial populations. Scraping uses sterile curettes to dislodge biofilm from deeper pockets, ensuring a more comprehensive sample of the microbial community. Paper points, which are absorbent, are inserted into peri-implant pockets to soak up crevicular fluid and the associated microorganisms. This technique is particularly useful for sampling from narrow and deep pockets where swabs or curettes may be less effective [14].

Timing and frequency are crucial in capturing the temporal dynamics of microbial communities. Sampling should ideally be performed at baseline (pre-treatment) and at various intervals post-treatment to monitor changes over time. Common intervals include one month, three months, and six months post-treatment, aligning with expected microbial shifts and clinical healing phases [11]. For example, in partially edentulous adults, an increased prevalence of red complex bacteria was observed one month post-treatment, followed by a decrease at three and six months. These temporal data are essential for understanding the progression or resolution of peri-implantitis at the microbiological level.

The proper handling and transport of samples are critical to prevent contamination and degradation. Samples should be collected using sterile techniques and immediately placed in transport media that preserve microbial viability. Common transport media include anaerobic transport medium and phosphate-buffered saline. Samples should be maintained at appropriate temperatures, usually between 2–8 °C, and transported to the laboratory as quickly as possible, ideally within 24 h [12]. Delays in transport can result in changes to the microbial community, potentially skewing results.

Ethical considerations in microbiological sampling are paramount. All procedures must comply with relevant guidelines and regulations, ensuring patient consent is obtained. Participants should be fully informed about the study’s purpose, procedures, and any potential risks. The study protocol should undergo review and approval by an ethical review board to ensure adherence to ethical standards [15].

### 2.2. Microbiological Analysis of Peri-Implant Microbiota

Microbiological analysis techniques described in peri-implant research range from traditional culture-based methods to advanced molecular approaches, each offering unique insights and advantages.

Culture-based methods involve growing microorganisms on selective media under controlled laboratory conditions. Specifically, culturing involves methods such as liquid cultures, which allow bacteria to grow in a nutrient-rich broth, and agar plates, where bacteria form visible colonies on solid media.

These methods are useful for isolating and identifying specific bacteria, particularly those that are present in low numbers. 

However, they are limited by the fact that not all bacteria can be cultured using standard techniques, leading to an underrepresentation of the microbial diversity present in the sample. This limitation is significant in environments like the oral cavity, where many bacteria are difficult to culture [16]. 

Molecular methods, including polymerase chain reaction (PCR) and next-generation sequencing (NGS), have revolutionized microbiology by sequencing DNA extracted from the sample and thus enabling the detection and identification of bacteria that cannot be cultured. 

PCR amplifies specific DNA sequences, allowing for the detection of particular bacteria, such as those in the red complex (*Porphyromonas gingivalis*, *Treponema denticola*, and *Tannerella forsythia*) associated with peri-implantitis [14]. 

NGS provides a comprehensive overview of the microbial community, offering insights into both the diversity and relative abundance of different bacterial species [17].

Quantitative PCR (qPCR) is utilized to quantify the amount of specific bacterial DNA in a sample, providing data on the bacterial load. It requires the development of standard curves using known quantities of target DNA to ensure accurate quantification.

This method is highly sensitive and specific, making it ideal for monitoring changes in the abundance of specific pathogens over time. qPCR is particularly useful in assessing the efficacy of peri-implantitis treatments by quantifying the reduction in pathogenic bacteria following therapy [18].

Metagenomics involves the direct sequencing of genetic material from environmental samples, bypassing the need for culturing. This approach offers a comprehensive picture of the microbial community, including rare and unculturable organisms. Indeed, this method has shown that cultivation-based techniques capture less than 1% of bacterial diversity in many samples.

Bioinformatics tools are then used to analyze the vast amounts of data generated, identifying species, predicting functions, and understanding interactions within the microbial community [19]. In peri-implantitis research, metagenomics can reveal shifts in microbial communities in response to treatment, highlighting potential biomarkers for disease progression or resolution.

## 3. Peri-Implant Microbiota

Understanding the microbial profile of peri-implant health and peri-implant mucositis- and peri-implantitis-associated microbiota involves examining the specific microorganisms that populate these sites [10,11,12,13,14,15,16,17,18,19,20], detailed in Table 1.

The predominant periodontal (and later peri-implant) pathogens have been classified based on their association with periodontal health or disease in so-called Socransky complexes [21,22]. Each of the red, orange, yellow, green, purple, and blue complex groups, named after the researcher S.S. Socransky, contains bacteria with similar pathogenic properties and roles in periodontal health or disease. Consequently, understanding these complexes helps in identifying the microbial profiles in different stages of periodontal and peri-implant disease.

The red complex is strongly linked to periodontal disease and peri-implantitis. Key members of this complex include *Porphyromonas gingivalis*, *Tannerella forsythia*, and *Treponema denticola*.

*Porphyromonas gingivalis* is a Gram-negative, anaerobic rod known for its virulence factors, such as proteases and fimbriae, which contribute to tissue destruction and immune evasion. It is consistently found in high levels in peri-implantitis sites, indicating its significant role in the disease [2,23,24,25].

*Tannerella forsythia*, another member of the red complex, is also a Gram-negative anaerobic bacterium that is key in severe periodontal disease. Its frequent identification in peri-implantitis sites underscores its contribution to the pathogenic profile of the disease [2,26].

*Treponema denticola*, a motile, anaerobic spirochete, is highly proteolytic and capable of tissue invasion, further contributing to the severity of peri-implantitis by degrading tissues and inducing inflammation [24,25].

The orange complex bacteria are associated with the progression of periodontal diseases and are often precursors to the red complex bacteria.

*Fusobacterium nucleatum*, a Gram-negative, anaerobic rod, serves as a bridge bacterium, facilitating the adhesion of other pathogens. It is identified in peri-implantitis and mucositis, playing a significant role in biofilm formation and disease progression [27].

Similarly, *Prevotella intermedia* and *Prevotella nigrescens*, both Gram-negative anaerobes, are involved in periodontal infections and are found in increased levels in peri-implantitis, contributing to the inflammatory response [28,29].

*Campylobacter rectus*, another member of the orange complex, is a Gram-negative, motile, curved rod associated with periodontal disease and detected in peri-implantitis sites, adding to the pathogenic microbial community [24].

*Parvimonas micra*, a Gram-positive, anaerobic coccus, also plays a role in peri-implantitis, being associated with disease progression [2].

The yellow complex bacteria are primarily health-associated, playing a crucial role in maintaining oral health. *Streptococcus* species, such as *Streptococcus sanguinis* and *Streptococcus gordonii*, are Gram-positive cocci that are early colonizers of dental biofilms. These bacteria are dominant in healthy peri-implant sites and contribute to a balanced microbial community [30].

Green complex bacteria, generally associated with health or early stage disease, include *Capnocytophaga* species. These Gram-negative rods are facultatively anaerobic and are less frequently associated with disease, found in lower levels in peri-implantitis [28].

The purple complex bacteria are also associated with periodontal health and are early colonizers of the biofilm. *Veillonella parvula*, a Gram-negative, anaerobic coccus, is involved in early biofilm formation and is present in healthy peri-implant sites, playing a role in maintaining microbial balance [29].

The blue complex bacteria, health-associated and involved in maintaining oral microbial homeostasis, include *Actinomyces* species such as *Actinomyces naeslundii*. These Gram-positive rods are facultatively anaerobic and are abundant in healthy peri-implant sites, contributing to biofilm stability and health [31].

Beyond the Socransky complexes, other microorganisms play significant roles in peri-implant health. *Rothia* species, which are Gram-positive cocci, are part of the normal oral flora and are found in higher proportions in healthy peri-implant sites, associated with oral health [30].

*Neisseria* species, Gram-negative diplococci that are part of the normal flora of the human mucosa, are more prevalent in healthy peri-implant sites, indicating their role in maintaining a balanced microbial community [28]. *Corynebacterium* species, Gram-positive rods that are facultatively anaerobic, are also found in healthy peri-implant sites, contributing to the stability of the microbial community [30].

*Methanobrevibacter oralis*, an archaeon involved in oral biofilms, is present in both healthy and diseased peri-implant sites but is not significantly associated with disease progression [2].

### 3.1. Microbial Profile of Healthy Peri-Implant Sites

Healthy peri-implant sites are characterized by a distinct microbiota that differs significantly from that found in diseased peri-implant tissues [32]. In detail, the microbial community of healthy peri-implant sites is typically less diverse and less pathogenic compared to that of peri-implantitis. Advanced sequencing techniques, such as 16S rRNA sequencing, have provided detailed insights into the composition of the microbiota in healthy peri-implant sites [33].

Studies utilizing 16S rRNA sequencing have shown that healthy peri-implant microbiota predominantly consist of Gram-positive facultative cocci and a low prevalence of periodontal pathogens. Mombelli et al. (1987) [31] and Leonhardt et al. (1999) [29] found that the microbiota of healthy implants was similar to that of healthy periodontal sites, with high proportions of coccoid cells, *Actinomyces*, and *Veillonella* species, and minimal anaerobic rods and fusobacteria [29,31,34].

More recent studies have confirmed these findings, showing that healthy peri-implant sites harbor a microbiota dominated by health-associated bacteria. For example, Zheng et al. (2015) [35] conducted a study using 16S rRNA sequencing to analyze the subgingival microbiome of healthy and ailing dental implants. They found that healthy implants were predominantly colonized by *Streptococci* and *Rothia*, which are associated with oral health [2,35]. Similarly, Sousa et al. (2017) [30] observed that healthy implants had a lower microbial diversity compared to diseased sites, with a dominance of Streptococci, suggesting a stable and health-associated microbial community [2,30].

Additionally, healthy implant sites are specifically associated with *Rothia*, *Neisseria*, and *Corynebacterium*, while diseased sites show a higher prevalence of pathogenic species such as *Porphyromonas gingivalis*, *Treponema denticola*, and *Tannerella forsythia* [2,30,36], which will be discussed later.

In healthy peri-implant sites, the microbial composition shows some overlap with that of periodontal sites but also distinct differences. Studies indicate that healthy peri-implant sulcus is predominantly colonized by bacteria from the phyla Firmicutes and Proteobacteria. Genera such as *Streptococcus* and *Neisseria* are abundant, reflecting a microbial environment that supports peri-implant health. *Peptostreptococcus micros*, although present, is typically found in lower abundance in healthy peri-implant sites compared to peri-implantitis sites [37].

Healthy periodontal sites typically exhibit a microbial community dominated by a diverse range of bacteria, primarily consisting of Gram-positive facultative anaerobes, including *Streptococcus* species and *Actinomyces* species. These bacteria play a crucial role in maintaining oral health by inhibiting the colonization of pathogenic species through competitive exclusion and the production of antimicrobial substances. Studies have shown that the microbial profiles of healthy peri-implant and periodontal sites are more similar to each other than to diseased sites, emphasizing the importance of maintaining a balanced microbial community to prevent disease onset. The comparison underscores the necessity for regular monitoring and early intervention to preserve the health of both periodontal and peri-implant tissues [37].

The stability and resilience of the microbial community in healthy peri-implant sites are critical for maintaining peri-implant health [38]. The presence of health-associated bacteria such as *Streptococcus* and *Actinomyces* plays a protective role by preventing the colonization and overgrowth of pathogenic species. These bacteria contribute to a balanced microbial ecosystem that supports tissue health and prevents inflammation. Healthy peri-implant sites also exhibit a lower microbial load and a less complex biofilm structure compared to diseased sites [39]. Studies have shown that the biofilm on healthy implants is less dense and composed of fewer species, predominantly Gram-positive cocci and rods. This contrasts with the biofilm in peri-implantitis, which is denser and more diverse, harboring a higher number of pathogenic species [37].

The differences in the microbial communities of healthy and diseased peri-implant sites highlight the importance of maintaining a stable and balanced microbiota for peri-implant health. Factors such as good oral hygiene practices, regular dental check-ups, and the use of antimicrobial agents can help maintain a healthy peri-implant microbiota and prevent the onset of peri-implant diseases [37].

**Figure 1:**
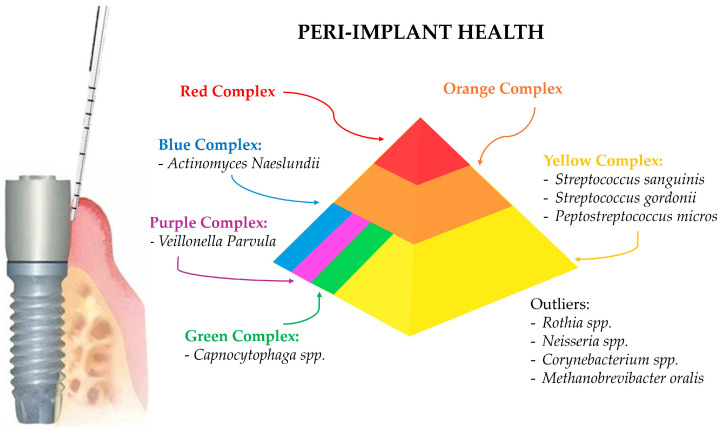
The healthy peri-implant site and the associated predominant microbial composition according to the Socransky complexes.

Figure 1 summarizes the predominant microbial composition in healthy peri-implant sites.

### 3.2. Peri-Implant Mucositis-Associated Microbiota

The microorganisms associated with peri-implant mucositis can be categorized according to the Socransky complexes, as already discussed, aiding in understanding their roles and associations with disease onset and progression.

The red complex bacteria, which are strongly associated with periodontal disease as well as peri-implantitis, are also found in peri-implant mucositis.

*Porphyromonas gingivalis* is known for its virulence factors such as proteases and fimbriae, which contribute to tissue destruction and immune evasion. This bacterium is detected in peri-implant mucositis, contributing to inflammation and the early stages of tissue damage [2,27,28,34].

*Tannerella forsythia* plays a significant role in the red complex due to its association with severe periodontal disease. It is found in peri-implant mucositis, indicating its involvement in the inflammatory process [2,27,28,34].

*Treponema denticola* is highly proteolytic and capable of tissue invasion. This bacterium is present in peri-implant mucositis, contributing to tissue degradation and inflammation [2,27].

The orange complex bacteria are involved in periodontitis progression and are also found in higher numbers in peri-implant mucositis.

In detail, *Fusobacterium nucleatum* serves as a bridge bacterium, facilitating the adhesion of other pathogens. It is found in peri-implant mucositis, playing a role in biofilm formation and early disease progression [2,27].

*Prevotella intermedia*, involved in various oral infections, including periodontitis, is detected in peri-implant mucositis, indicating its contribution to the inflammatory response [2,27,28,34].

*Campylobacter rectus* is associated with periodontal disease and identified in peri-implant mucositis, contributing to the pathogenic microbial community [2,27].

*Parvimonas micra* is known for its role in periodontal infections and is found in peri-implant mucositis, associated with early disease stages [2,27].

The yellow complex bacteria are primarily health-associated but can be found in peri-implant mucositis as well. *Streptococcus* species, such as *Streptococcus sanguinis* and *Streptococcus gordonii*, are early colonizers of periodontal and peri-implant biofilms and important for maintaining oral health. These bacteria are present in both healthy and peri-implant mucositis sites, suggesting their transitional role in the shift from health to disease [2,30].

The green complex bacteria are generally associated with health or early stage disease. *Capnocytophaga* species are found in peri-implant mucositis, though in lower abundance compared to more pathogenic species [28,34].

The purple complex bacteria are associated with periodontal health and are early colonizers of the biofilm. *Veillonella parvula* is involved in early biofilm formation and present in peri-implant mucositis, contributing to the complexity of the biofilm [29,34].

The blue complex bacteria are health-associated and involved in maintaining oral microbial homeostasis. *Actinomyces* species, such as *Actinomyces naeslundii*, are found in peri-implant mucositis, indicating their role in biofilm stability and early disease stages [31,34]

Beyond the Socransky complexes, other microorganisms play significant roles in peri-implant mucositis. *Rothia* species, which are part of the normal oral flora, are present in peri-implant mucositis, suggesting a role in the transition from health to disease [2,30].

*Neisseria* species, also belonging to the normal flora of the human mucosa, are more prevalent in peri-implant mucositis compared to healthy sites, indicating their involvement in early inflammation [28,34].

*Corynebacterium* species are facultatively anaerobic and are found in peri-implant mucositis, contributing to microbial diversity and early disease processes [2,30]. *Methanobrevibacter oralis*, an archaeon involved in oral biofilms, is present in both healthy and peri-implant mucositis sites but is not significantly associated with disease progression [34].

**Figure 2:**
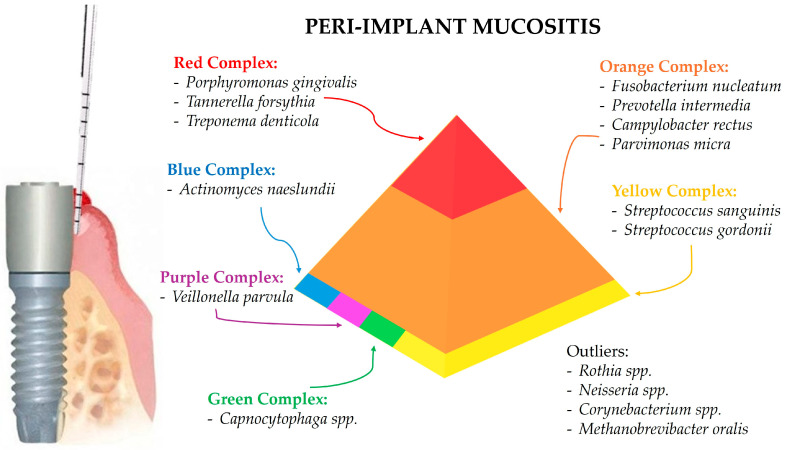
The peri-implant mucositis site and the associated predominant microbial composition according to the Socransky complexes.

Figure 2 summarizes the predominant microbial composition in peri-implant mucositis sites.

### 3.3. Peri-Implantitis-Associated Microbiota

The microbial profile of peri-implantitis is highly diverse and includes a wide range of pathogenic bacteria. Advanced sequencing techniques have provided detailed insights into the complex and diverse microbial communities associated with peri-implantitis.

The increased microbial diversity and pathogenicity observed in peri-implantitis are associated with the dysbiosis of the microbial community [40]. Dysbiosis refers to the imbalance between beneficial and harmful microorganisms, leading to a state where pathogenic species dominate and contribute to disease progression [41]. In peri-implantitis, dysbiosis results in the overgrowth of pathogenic bacteria, increased inflammation, and tissue destruction. Such a microbial variety is significantly higher compared to healthy and peri-implant mucositis sites. This increased diversity is associated with a more complex and pathogenic biofilm structure. Studies have shown that the biofilm in peri-implantitis includes a higher number of species and a greater abundance of pathogens compared to healthy sites. The presence of diverse microbial communities, including less commonly identified species such as *Staphylococcus* and *Enterobacteriaceae*, underscores the complexity of the disease and the need for comprehensive microbiological assessments [2,24,34].

Studies using 16S rRNA sequencing have shown that peri-implantitis is associated with a high prevalence of Gram-negative anaerobes, which are also commonly found in periodontitis. Key pathogens identified in peri-implantitis include *Porphyromonas gingivalis*, *Tannerella forsythia*, and *Treponema denticola*, which are part of the red complex and are known for their role in periodontal diseases [2,24,34]. On the other hand, the presence of unique pathogens such as *Filifactor alocis* and *Fretibacterium fastidiosum* further highlights the complexity of the microbial community in peri-implantitis.

The red complex bacteria are most strongly associated with severe periodontal diseases and peri-implantitis. These are considered predominant pathogens due to their virulence factors and ability to induce a strong inflammatory response. These bacteria can evade the host immune system, produce toxins, and degrade host tissues, contributing to the severity of peri-implantitis.

Indeed, *Porphyromonas gingivalis* is consistently found in high levels in peri-implantitis sites, indicating its substantial role in peri-implantitis pathogenesis [2,24,34].

The frequent identification of *Tannerella forsythia* in peri-implantitis sites further underscores its pathogenic contribution [2,26,34].

*Treponema denticola* contributes significantly to the tissue degradation and inflammation seen in peri-implantitis [2,24].

The orange complex bacteria are associated with the progression of periodontal diseases and are also prevalent in peri-implantitis. *Fusobacterium nucleatum* is identified in peri-implantitis, playing a significant role in biofilm formation and disease progression [2,27].

*Prevotella intermedia* is found in increased levels in peri-implantitis, contributing to the inflammatory response [28,34].

Similarly, *Prevotella nigrescens* is often found in conjunction with other periodontal pathogens in peri-implant diseases [29,34].

*Campylobacter rectus* is generally detected in peri-implantitis sites, adding to the pathogenic microbial community [2,24].

Additionally, *Parvimonas micra* is frequently associated with peri-implantitis, indicating its role in early disease stages [34].

Comparing the microbial profiles of peri-implantitis and periodontitis sites, there is a notable overlap in the presence of key pathogens such as *Porphyromonas gingivalis*, *Tannerella forsythia*, and *Treponema denticola*. However, studies have shown that peri-implantitis sites tend to harbor a higher diversity of pathogenic bacteria, including unique species like *Filifactor alocis* and *Fretibacterium fastidiosum*, which are not commonly found in periodontitis sites [2,26]. Additionally, peri-implantitis sites show a higher prevalence of Gram-negative anaerobes and other opportunistic pathogens like *Staphylococcus* and *Enterobacteriaceae*, which contribute to the complex biofilm and disease progression [2,24,34].

However, in addition to these well-known periodontal pathogens, peri-implantitis is characterized by the presence of unique bacterial species that are not commonly found in periodontitis. For example, Sanz-Martin et al. (2017) [26] identified new pathogens specific to peri-implantitis, such as *Filifactor alocis* and *Fretibacterium fastidiosum*. These findings suggest that while there is an overlap in the microbial communities of periodontitis and peri-implantitis, the latter harbors unique bacterial species that contribute to its pathogenesis [2,26].

Overall, the microbial profile of peri-implantitis demonstrates a complex interplay of various pathogenic and health-associated bacteria, highlighting the importance of advanced microbiological techniques in understanding the disease. Identifying these microorganisms and understanding their roles in peri-implantitis can lead to better preventive and therapeutic strategies, ultimately improving patient outcomes.

Accordingly, a deeper knowledge of the microbial diversity and pathogenicity in peri-implantitis is crucial for developing effective treatment strategies [42]. Traditional approaches to managing peri-implantitis, such as mechanical debridement and the use of antibiotics [43], may not be sufficient to address the complex microbial communities involved. Advanced therapies targeting specific pathogens and restoring microbial balance (reversing oral dysbiosis) are needed [44] to effectively treat peri-implantitis and prevent implant failure.

**Figure 3:**
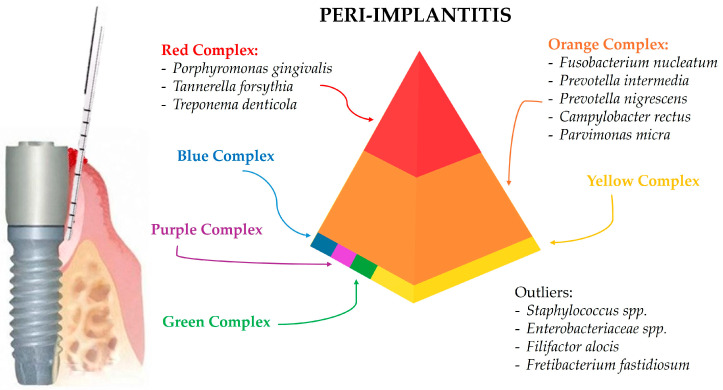
The peri-implantitis site and the associated predominant microbial composition according to the Socransky complexes.

Figure 3 summarizes the predominant microbial composition in peri-implantitis sites.

#### 3.3.1. *Candida albicans* in Peri-Implantitis

*Candida albicans* is a fungal pathogen commonly implicated in various oral infections, including peri-implantitis. This opportunistic yeast can adhere to and colonize both mucosal surfaces and inert materials and is capable of biofilms, which is a critical factor in its pathogenicity. Indeed, biofilms protect *Candida albicans* from environmental stresses, including antifungal agents and the host immune response [45].

*Candida albicans* biofilms are particularly problematic because they provide a protective environment not only for the yeast itself but also for other microbial inhabitants, including bacterial pathogens. The biofilm matrix, composed of extracellular polymeric substances, shields the microbial community from antimicrobial agents and the host immune system. This environment facilitates the survival and proliferation of both fungi and bacteria, complicating treatment efforts and leading to chronic infection [46].

In the context of peri-implantitis, *Candida albicans* can adhere to implant surfaces and form biofilms that harbor a complex community of microorganisms, contributing to the persistence and severity of the infection [47,48,49].

Studies have demonstrated that the presence of *Candida albicans* in peri-implantitis sites is often associated with increased microbial diversity and higher counts of predominant pathogens, exacerbating the inflammatory response and tissue destruction [47,50].

The involvement of *Candida albicans* in peri-implantitis also highlights the importance of comprehensive antimicrobial strategies that address both bacterial and fungal components of the infection. Coherently, traditional treatments focused solely on bacterial pathogens may be insufficient in the presence of *Candida albicans*, necessitating the use of antifungal agents and biofilm-disrupting therapies to effectively manage the infection [51]. Hence, understanding the role of C. albicans in peri-implantitis underscores the need for a multifaceted approach to treatment, targeting the diverse microbial communities that contribute to the disease’s progression [47,49].

#### 3.3.2. Herpes Simplex Virus (HSV) in Peri-Implantitis

Herpes Simplex Virus (HSV), particularly HSV-1, is a common viral pathogen that has been implicated in peri-implantitis. HSV-1 is known for its ability to establish latent infections in sensory ganglia and reactivate periodically, causing lesions at the site of infection. In the oral cavity, HSV-1 can infect epithelial cells and contribute to the development and exacerbation of peri-implantitis by promoting an environment conducive to bacterial colonization and biofilm formation [47,48,50]. Indeed, the presence of HSV-1 in peri-implantitis sites has been associated with higher levels of periopathogenic bacteria, such as *Porphyromonas gingivalis*, *Treponema denticola*, and *Tannerella forsythia*.

This association suggests a synergistic relationship between viral and bacterial pathogens, where HSV-1 may enhance the pathogenic potential of these bacteria. The virus can induce changes in the local immune response, reducing the efficacy of the host’s defenses and allowing bacteria to thrive.

Additionally, HSV-1 infection can lead to the production of pro-inflammatory cytokines, which contribute to tissue inflammation and destruction, key features of peri-implantitis [47,52].

Research indicates that HSV-1 may also disrupt the integrity of the epithelial barrier, making it easier for bacterial pathogens to invade deeper tissues. This disruption, coupled with the virus’s ability to evade immune detection and persist in a latent state, complicates the clinical management of peri-implantitis.

As a result, it may be hypothesized that effective treatment may require antiviral therapies, in addition to conventional surgical and nonsurgical mechanical debridement, to address both the viral and bacterial components of the infection [47,48,50].

#### 3.3.3. Epstein–Barr Virus (EBV) in Peri-Implantitis

Epstein–Barr Virus (EBV), a member of the herpesvirus family, is another viral pathogen associated with peri-implantitis. EBV is known for its ability to establish latent infections in B lymphocytes and epithelial cells, with periodic reactivations. In the context of peri-implantitis, EBV has been detected at higher levels in peri-implant lesions compared to healthy implant sites, indicating its potential role in the disease’s pathogenesis [47,52].

The involvement of EBV in peri-implantitis is thought to be multifactorial. EBV can modulate the host immune response, leading to a localized immunosuppressive environment that favors bacterial colonization and biofilm formation.

Similar to HSV-1, EBV infection can promote an inflammatory milieu through the induction of cytokines and other inflammatory mediators. This inflammation can enhance the pathogenic potential of co-infecting bacteria, exacerbating tissue destruction and bone loss characteristics of peri-implantitis [47,50].

Moreover, EBV’s ability to persist in a latent state within host cells poses a challenge for treatment. Latent EBV can evade immune detection and reactivate under certain conditions, contributing to the chronic nature of peri-implantitis.

For these reasons, an effective management of peri-implantitis in the presence of EBV may require antiviral strategies alongside traditional periodontal therapies to target both the viral and bacterial components of the disease [47,52].

## 4. Discussion

This narrative review aimed to analyze the microbial profiles associated with healthy peri-implants and peri-implant mucositis- and peri-implantitis-associated microbiota and the related microbiological sampling and analyses.

By comparing microbiologic outcomes from healthy and diseased implants in various studies, it has been possible to identify key microorganisms linked to peri-implantitis. A comprehensive examination of the microbiologic profile associated with peri-implantitis reveals significant insights into the disease’s etiology and related management.

The advent of NGS has revolutionized the understanding of microbial communities in peri-implant infections. NGS methods, particularly 16S ribosomal RNA gene amplicon sequencing, have revealed distinct microbiotas associated with peri-implant health and disease, indicating that peri-implantitis has a differentially abundant microbiota compared to periodontitis [53]. Studies have shown that peri-implantitis is associated with an increase in microbial diversity and a shift towards a more pathogenic community, including classical and emerging periodontal pathogens [23].

### 4.1. Microbial Profile of Healthy Peri-Implant Sites

Upon implant placement, a salivary pellicle rapidly forms on the exposed surfaces, facilitating early bacterial colonization. Early bacterial communities can achieve a symbiotic equilibrium with the host and maintain clinical (macroscopic) peri-implant health, apart from the low-level paraphysiologic reactive inflammatory infiltrate [4], as in periodontal tissues. Histologically, peri-implant tissues show distinct differences compared to natural teeth. Natural teeth are anchored via the periodontal ligament (PDL), whereas dental implants are osseointegrated, meaning they are directly anchored to the bone without PDL. This lack of PDL limits the blood supply, thereby restricting the amount of nutrients and immune cells available to tackle early bacterial infections [3,4]. Moreover, fibers of the supracrestal connective tissue are positioned circumferentially around implants rather than perpendicularly, which reduces the physical barrier against bacterial invasion into the submucosa, placing peri-implant tissues in a more vulnerable “open wound” state [4].

In healthy peri-implant sites, the microbial composition overlaps somewhat with that of periodontal sites [53], which is conceivable considering that the periodontal niche may serve as a reservoir for periodontopathogens in dentate subjects [54], yet distinct differences are also observed. However, the implant surface structure and abutment interface significantly influence microbial colonization and disease progression. Modifying the surface characteristics can enhance antimicrobial properties and clinical outcomes [3]. The prolonged exposure of the metal surface to biofilm or friction at the implant–abutment interface can lead to biocorrosion, releasing titanium particles and potentially causing an inflammatory response in surrounding tissues [53]. While most studies focus on titanium implants, zirconia implants have also been examined, with varying results regarding microbial colonization and peri-implant health [23]. These implant-centered factors contribute to the differences observed between peri-implant and periodontal diseases.

Healthy periodontal sites generally host a diverse bacterial community, primarily composed of Gram-positive facultative anaerobes such as *Streptococcus* and *Actinomyces* species. These bacteria are essential for peri-implant health, as they prevent the colonization of pathogenic species through competitive exclusion and the production of antimicrobial substances. However, factors that promote biofilm growth can lead to tissue inflammation, altering the peri-implant sulcus microenvironment and causing dysbiotic shifts in the microbiota that exacerbate inflammatory progression [3].

### 4.2. Peri-Implant Mucositis-Associated Microbiota

The progression from health to peri-implantitis involves a series of microbial shifts [4,55,56,57,58].

In peri-implant mucositis, various microorganisms play distinct roles, reflecting their involvement in the transition from health to disease. The red complex bacteria, strongly associated with periodontal disease and peri-implantitis, are also found in peri-implant mucositis. Similarly, the orange complex bacteria, involved in periodontitis progression, are present in higher numbers in peri-implant mucositis [58].

Although the yellow complex bacteria are primarily health-associated, they can also be found in peri-implant mucositis. Indeed, *Streptococcus* species, early colonizers of periodontal and peri-implant biofilms crucial for maintaining oral health, are present in both healthy and peri-implant mucositis sites, suggesting their transitional role in the shift from health to disease. The purple complex bacteria, including *Veillonella parvula*, are linked to periodontal health and are early colonizers of the biofilm. Similarly, the blue complex bacteria, such as *Actinomyces* species, are health-associated and contribute to maintaining oral microbial homeostasis [4,55,56,57,58].

The green complex bacteria are generally associated with health or early stage disease, such as *Capnocytophaga*.

In the same way, beyond the Socransky complexes, *Rothia* species, part of the normal oral flora, are present in peri-implant mucositis, suggesting a role in the transition from health to disease.

*Neisseria* species, Gram-negative diplococci part of the normal flora of the human mucosa, are more prevalent in peri-implant mucositis compared to healthy sites, indicating their involvement in early inflammation, as *Corynebacterium* species, part of the normal flora, contribute to this inflammatory process.

As the disease progresses, there is an increase in microbial diversity and a shift towards pathogens associated with increased proinflammatory cytokines [4]. Smokers exhibit lower microbial diversity and higher abundances of disease-associated species compared to non-smokers, with significant changes observed during the transition from health to peri-implant mucositis and then to peri-implantitis [3].

### 4.3. Peri-Implantitis-Associated Microbiota

Peri-implantitis is considered an endogenous mixed infection, occasionally involving nontypical oral bacteria [4]. The primary risk factors include poor oral hygiene, smoking, and a history of periodontitis [4].

In peri-implantitis, a significantly higher microbial diversity compared to healthy and peri-implant mucositis sites is observed, associated with a more complex and pathogenic biofilm structure. A body of evidence identifies a variety of microorganisms associated with peri-implantitis, including 6 bacterial phyla, 17 bacterial genera, 23 bacterial species, and 2 genera of viruses. Among these, *Porphyromonas gingivalis*, *Treponema denticola*, and *Tannerella forsythia* have emerged as the main bacterial species associated with peri-implantitis.

Red complex species are found in significantly higher counts in peri-implantitis compared to healthy peri-implant sites [59,60,61,62]. Additionally, there is some evidence supporting the association of *Prevotella intermedia* and *Campylobacter rectus* with peri-implantitis [59,60,61]. Indeed, the orange complex bacteria, including *Fusobacterium nucleatum* and *Prevotella intermedia*, are also prevalent in peri-implantitis, contributing to biofilm formation, disease progression, and the inflammatory response. Additionally, peri-implantitis sites harbor unique bacterial species like *Filifactor alocis* and *Fretibacterium fastidiosum*, which are not commonly found in periodontitis, suggesting an overlapping yet distinct microbial community between the two conditions. These specific pathogens are Gram-negative, anaerobic bacteria previously linked to periodontitis, suggesting a connection between the two conditions [63]. This association indicates that the periodontal and peri-implant environments share similar microbial ecosystems, reinforcing the notion that a history of periodontitis may increase the risk of peri-implantitis [21,64].

Since the ecological and functional characteristics of the peri-implant niche, influenced by the implant surface, play a crucial role in the pathogenesis of peri-implant infections, understanding these dynamics is essential for developing improved strategies for the prevention, diagnosis, and treatment of peri-implant diseases. The application of high-throughput sequencing technologies continues to expand the knowledge of peri-implant microbiota, highlighting the importance of targeting microbial communities and their functional pathways in managing peri-implant infections [4]. In detail, the functional assessment of microbial communities in peri-implantitis, assessing the transcriptome of the associated bacteria (metatranscriptomic analysis) by sequencing total RNA from microbial communities, has shown that peri-implantitis and periodontitis share similar virulence factors but differ in their interaction networks and functional profiles [53], potentially suggesting that peri-implantitis is driven by the functional pathogenicity of the microbial community rather than the presence of specific pathogenic taxa [3]. Accordingly, it has been proposed that, as bacterial invasion is crucial in infection, understanding the differentiation of *Prevotella intermedia* in peri-implantitis sites is crucial. Indeed, *Prevotella intermedia* 17 exhibits type C fimbriae, distinguishing it from other strains such as *Prevotella intermedia* 27 (type D) and *Prevotella intermedia* 25611 (type A) [65]. Type C fimbriae, along with cytoskeletal rearrangements, significantly enhance the ability of *Prevotella intermedia* 17 to be internalized by oral epithelial cells compared to other strains [65]. Similarly, *Porphyromonas gingivalis*, facilitated by its large fimbriae, which bind to β1 integrin on host cell surfaces, causing actin cytoskeleton rearrangements for internalization [66], also invades gingival epithelial cells to evade immune detection and replicate [66]. Within host cells, *Porphyromonas gingivalis* secretes an ATP-hydrolyzing enzyme to prevent apoptosis or necrosis, ensuring intracellular survival [66], and can also spread from cell to cell without inducing cell death, further evading the immune system [66].

Research has also explored the potential role of viruses in peri-implantitis. Studies have found a higher prevalence of human herpesvirus 4 (Epstein–Barr virus) and human cytomegalovirus in peri-implantitis compared to healthy implants [67,68]. However, the evidence is not robust enough to establish a definitive role for these viruses in the disease’s etiology.

Moreover, the involvement of *Candida albicans* in peri-implantitis, similar to HSV and EBV, highlights the complexity of the disease’s etiology. These pathogens may contribute to the persistence and severity of the infection through biofilm formation, immune modulation, and synergistic interactions with bacterial pathogens.

Consequently, an effective management of peri-implantitis requires a multifaceted approach that addresses both the fungal and viral components of the infection, in addition to the bacterial factors. Understanding these interactions is essential for developing comprehensive diagnostic and therapeutic strategies to improve patient outcomes [69].

Despite these findings, several limitations exist within the research. A relevant heterogeneity was found among the studies, including differences in microbial detection methods and molecular techniques used for microbial identification varying in sensitivity and specificity, potentially affecting the interpretation of data [49,52] and data presentation. In addition, the lack of standardized diagnostic criteria for peri-implantitis across studies leads to variability in prevalence rates and complicates comparisons in the original studies as well as systematic reviews including them. Most studies were cross-sectional, providing a snapshot of microbial profiles at a single point in time, which limits the ability to establish temporal relationships between microbial shifts and disease progression [49,61]. Furthermore, the inconsistent exclusion of confounding factors, such as smoking and systemic health conditions, further impacts study results [47]. Indeed, some studies also involved smokers in the peri-implantitis group, which could influence the composition of the oral microbiota and confound the results [70,71,72].

The need for comprehensive microbial data is emphasized, including levels of microorganisms to better understand their pathogenic potential. Accordingly, establishing causation remains challenging, as it has been proposed that the presence of these microorganisms in peri-implantitis could result from the disease environment rather than being the cause [47].

To address these limitations, future research should aim to standardize diagnostic criteria for peri-implantitis and adopt consistent sampling methods. Utilizing advanced molecular techniques, such as metagenomics and next-generation sequencing, can provide a more comprehensive understanding of the peri-implant microbiome. These techniques can detect a broader range of microorganisms, including unculturable and novel species, and offer insights into microbial interactions and functional potential [49,61].

Integrative approaches that combine microbial data with host immune response profiles and clinical outcomes could elucidate the complex interplay between the host and microbiota in peri-implantitis. This holistic view can help identify biomarkers for early detection and targets for therapeutic interventions. Understanding the microbial and host factors contributing to peri-implantitis can pave the way for personalized treatment strategies, tailoring interventions based on individual microbial profiles and immune responses to enhance treatment efficacy and prevent disease recurrence [64].

Clinically, the identification of predominant pathogens associated with peri-implantitis may allow for the development of microbial risk assessment tools, which may help clinicians identify patients at higher risk for peri-implantitis and implement preventive measures or early interventions.

Similarly, the regular monitoring of microbial profiles before, during, and after treatment can help assess the efficacy of therapeutic interventions and guide adjustments in treatment plans [73] to ensure the eradication of pathogenic microorganisms and the maintenance of a healthy peri-implant environment [74,75,76,77].

Moreover, a knowledge of microbial profiles can inform the selection of targeted antimicrobial therapies, improving treatment outcomes by focusing on specific pathogens [47].

## 5. Conclusions

Robust sampling techniques, coupled with advanced molecular and bioinformatics tools, may provide detailed insights into the microbial dynamics involved in peri-implantitis etiology and guide preventive and treatment strategies.

Compared to healthy periodontal sites, which typically exhibit a diverse microbial community dominated by Gram-positive facultative anaerobes, the microbial profile of healthy peri-implant sites is predominantly composed of Gram-positive cocci and facultatively anaerobic rods and constitutes a stable community that prevents the colonization and overgrowth of pathogenic species, thus being essential for maintaining peri-implant health and preventing the onset of peri-implant diseases.

The presence of red and orange complex bacteria in peri-implant mucositis-associated microbiota indicates a shift towards a more pathogenic state, contributing to inflammation and early tissue damage. However, various microorganisms play key roles in the transition from health to disease. Yellow complex bacteria, although primarily health-associated, and *Streptococcus* species, early colonizers crucial for oral health, are also present in both healthy and mucositis sites, along with green, purple, and blue complex bacteria, linked to health or early stage disease. Additionally, *Rothia*, *Neisseria*, and *Corynebacterium* species, part of the normal oral flora, play significant roles in the inflammation characteristics of peri-implant mucositis. Understanding these microbial communities may be essential for early detection and intervention in peri-implant diseases.

In peri-implantitis, there is a significantly higher microbial diversity compared to healthy and peri-implant mucositis sites, resulting in a more complex and pathogenic biofilm structure. Predominant pathogens include *Porphyromonas gingivalis*, *Tannerella forsythia*, *Treponema denticola*, and *Fusobacterium nucleatum*, along with unique species like *Filifactor alocis* and *Fretibacterium fastidiosum*. Additionally, peri-implantitis sites harbor less common species such as *Staphylococcus* and *Enterobacteriaceae*, contributing to disease progression through a complex biofilm and increased inflammatory response.

Research has also revealed a higher prevalence of EBV, human cytomegalovirus, and *Candida albicans* in peri-implantitis compared to healthy sites, probably contributing to the persistence and severity of the disease.

## Figures and Tables

**Table 1:** Microorganisms at healthy and pathological peri-implant sites in alphabetical order: bacteria; genus; phylogenetic tree; features; adherence and toxin production; antimicrobial susceptibility; antibiotic resistance; (putative) role in peri-implant health and diseases.

Bacteria	Genus	Phylogenetic Tree	Features	Adherence and Toxin Production	Antimicrobial Susceptibility	Antibiotic Resistance	Role in Peri-Implant Health and Diseases
*Actinomyces naeslundii*	Genus Actinomyces, family Actinomycetaceae	Closely related to Actinomyces oris and Actinomyces johnsonii	Anaerobic or microaerophilic, Gram-positive, rod-shaped, non-spore forming	Adheres to oral surfaces, contributing to initial biofilm formation	Susceptible to penicillin and other beta-lactam antibiotics	Generally low, but resistance can occur via beta-lactamase production	Associated with good oral health; early colonizer of dental biofilms, contributes to biofilm stability.
*Campylobacter rectus*	Genus Campylobacter, family Campylobacteraceae	Initially classified as Wolinella recta, reclassified based on rRNA analysis	Facultative anaerobe, Gram-negative, rod-shaped, motile	Adheres to periodontal tissues, forms biofilms, moves using a single flagellum	Susceptible to multiple antibiotic classes, including macrolides and beta-lactams	MD	Detected in peri-implant mucositis and peri-implantitis, contributing to the pathogenic microbial community.
*Capnocytophaga*	Genus Capnocytophaga, family Flavobacteriaceae	Part of the family Flavobacteriaceae	Capnophilic, anaerobic, Gram-negative, fusiform bacilli	Adheres to oral tissues, uses gliding motility for movement	Generally susceptible to antibiotics	Beta-lactam resistance due to beta-lactamase production	Generally associated with health or early stage disease; found in lower abundance in peri-implant mucositis.
*Corynebacterium*	Genus Corynebacterium, family Corynebacteriaceae	Related to genera like Mycobacterium and Streptomyces	Aerobic, some facultatively anaerobic, Gram-positive, rod-shaped	Produces toxins, adheres to epithelial cells	Susceptible to a range of antibiotics	Resistance varies, some strains producing beta-lactamase	Part of normal oral flora; found in healthy peri-implant sites and peri-implant mucositis, contributing to microbial diversity.
*Enterobacteriaceae*	Large family including genera like Escherichia and Salmonella	Part of the order Enterobacterales, class Gammaproteobacteria	Facultative anaerobes, Gram-negative, rod-shaped	Adheres to intestinal cells using fimbriae, some produce exotoxins	Varies widely among species	Common, with many strains producing beta-lactamase or other mechanisms	Includes opportunistic pathogens found in peri-implantitis; contribute to complex biofilm and disease progression.
*Fusobacterium nucleatum*	Genus Fusobacterium, family Fusobacteriaceae	Part of the phylum Fusobacteriota	Obligate anaerobe, Gram-negative, rod-shaped	Adheres to gingival and other epithelial cells, invades endothelial cells	Generally susceptible to metronidazole and beta-lactams	Resistance to some antibiotics reported	Found in peri-implant mucositis and peri-implantitis, plays a significant role in biofilm formation and disease progression.
*Neisseria*	Genus Neisseria, family Neisseriaceae	Part of the family Neisseriaceae within the phylum Proteobacteria	Aerobic, oxidase-positive, Gram-negative, diplococci	Adheres to mucosal surfaces using pili, produces LOS (lipooligosaccharide)	Varies, some strains showing resistance	Resistance to penicillin, ciprofloxacin, and others due to beta-lactamase	Abundant in healthy peri-implant sites; more prevalent in peri-implant mucositis, indicating involvement in early inflammation.
*Parvimonas micra*	Genus Parvimonas, family Peptostreptococcaceae	Related to other anaerobic Gram-positive cocci	Anaerobic, Gram-positive, coccus-shaped	Adheres to oral and systemic tissues, contributing to abscess formation	Generally susceptible to beta-lactam antibiotics	MD	Frequently associated with peri-implantitis and peri-implant mucositis, involved in early disease stages.
*Peptostreptococcus*	Genus Peptostreptococcus, family Peptostreptococcaceae	Related to other anaerobic Gram-positive cocci	Anaerobic, Gram-positive, small spherical cells	Can form part of mixed infections	Susceptible to beta-lactam antibiotics	Increasing resistance to antimicrobial drugs reported	Present in lower abundance in healthy peri-implant sites; typically found in higher levels in peri-implantitis sites.
*Porphyromonas gingivalis*	Genus Porphyromonas, family Porphyromonadaceae	Part of the Bacteroidota phylum	Anaerobic, Gram-negative, rod-shaped	Adheres to gingival epithelial cells, invades host cells, forms biofilms	Susceptible to metronidazole and other antibiotics	MD	Strongly associated with peri-implantitis; found in peri-implant mucositis, contributing to inflammation and tissue damage.
*Prevotella intermedia*	Genus Prevotella, family Prevotellaceae	Part of the Bacteroidota phylum	Anaerobic, Gram-negative, rod-shaped	Adheres to oral tissues, contributing to inflammation and tissue destruction	Susceptible to various antibiotics	MD	Detected in peri-implant mucositis and peri-implantitis, contributes to inflammatory response.
*Prevotella nigrescens*	Genus Prevotella, family Prevotellaceae	Part of the Bacteroidota phylum	Anaerobic, Gram-negative, rod-shaped	Adheres to oral tissues, contributing to inflammation and disease	Susceptible to various antibiotics	MD	Often found in peri-implant diseases, triggers immune responses leading to periodontal disease.
*Rothia*	Genus Rothia, family Micrococcaceae	Part of the family Micrococcaceae	Aerobic, Gram-positive, rod-shaped, non-motile	Adheres to oral and gut tissues	Generally susceptible to antibiotics	MD	Associated with oral health; present in healthy peri-implant sites and peri-implant mucositis, suggests role in transition from health to disease.
*Treponema denticola*	Genus Treponema, family Spirochaetaceae	Part of the Spirochaetes phylum, closely related to Treponema pallidum	Anaerobic, Gram-negative, spirochete, motile	Adheres to gingival fibroblasts, invades host cells, produces cytotoxic effects	Generally susceptible to antibiotics	MD	Highly proteolytic; part of the red complex, associated with peri-implant mucositis and peri-implantitis.
*Tannerella forsythia*	Genus Tannerella, family Bacteroidaceae	Part of the Bacteroidota phylum	Anaerobic, Gram-negative, rod-shaped	Adheres to periodontal tissues, contributing to inflammation and tissue destruction	Generally susceptible to antibiotics	MD	Part of the red complex; found in peri-implant mucositis and peri-implantitis, involved in inflammatory process.
*Veillonella parvula*	Genus Veillonella, family Veillonellaceae	Part of the Negativicutes class within the Firmicutes phylum	Anaerobic, Gram-negative, coccus-shaped	Adheres to oral tissues, forms biofilms with Streptococcus species	Susceptible to metronidazole, penicillin, cephalosporins, clindamycin, and chloramphenicol	Reports of resistance to various antibiotics in different countries	Part of normal oral flora; involved in early biofilm formation, present in peri-implant mucositis.

Acronym: missing data, “MD”.
